# Risk of latent TB infection in individuals employed in the healthcare sector in Germany: a multicentre prevalence study

**DOI:** 10.1186/1471-2334-10-107

**Published:** 2010-04-30

**Authors:** Anja Schablon, Melanie Harling, Roland Diel, Albert Nienhaus

**Affiliations:** 1 Institution for Statutory Accident Insurance and Prevention in the Health and Welfare Services, Department of Occupational Health Research, Pappelallee 35-37, 22089 Hamburg, Germany; 2 University Medical Center Hamburg-Eppendorf, Institute for Health Services Research in Dermatology and Nursing, Martinistraße 52, 20246 Hamburg, Germany; 3 Department of Pulmonary Medicine, Hannover Medical School (MHH), Carl-Neuberg-Straße1, 30625 Hannover, Germany

## Abstract

**Background:**

Healthcare workers are still recognised as a high-risk group for latent TB infection (LTBI). Therefore, the screening of people employed in the healthcare sector for active and LTBI is fundamental to infection control programmes in German hospitals. It was the aim of the study to determine the prevalence and putative risk factors of LTBI.

**Methods:**

We tested 2028 employees in the healthcare sector with the QuantiFERON-Gold In-tube (QFT-IT) test between December 2005 and May 2009, either in the course of contact tracing or in serial testing of TB high-risk groups following German OSH legislation.

**Results:**

A positive IGRA was found in 9.9% of the healthcare workers (HCWs). Nurses and physicians showed similar prevalence rates (9.7% to 9.6%). Analysed by occupational group, the highest prevalence was found in administration staff and ancillary nursing staff (17.4% and 16.7%). None of the individuals in the trainee group showed a positive IGRA result. In the different workplaces the observed prevalence was 14.7% in administration, 12.0% in geriatric care, 14.2% in technicians (radiology, laboratory and pathology), 6.5% in admission ward staff and 8.3% in the staff of pulmonary/infectious disease wards. Putative risk factors for LTBI were age (>55 years: OR14.7, 95% CI 5.1-42.1), being foreign-born (OR 1.99, 95% CI 1.4-2.8), TB in the individual's own history (OR 4.96, 95% CI 1.99-12.3) and previous positive TST results (OR 3.5, 95% CI 2.4-4.98). We observed no statistically significant association with gender, BCG vaccination, workplace or profession.

**Conclusion:**

The prevalence of LTBI in low-incidence countries depends on age. We found no positive IGRA results among trainees in the healthcare sector. Incidence studies are needed to assess the infection risk. Pre-employment screening might be helpful in this endeavour.

## Background

The risk of latent tuberculosis infection (LTBI) and active tuberculosis (TB) as an occupational disease is well established and healthcare workers (HCWs) are still recognised as a high-risk group for LTBI [1-3]. Therefore, the screening of individuals employed in the healthcare sector for active TB and LTBI is fundamental to infection control programmes in hospitals, especially in low-incidence countries like Germany with a TB incidence of 6.1/100.000 in 2007 [4,5]. In accordance with the German guidelines of occupational safety and health legislation (OSH) HCWs are screened routinely depending on risk assessment. All HCWs working in pulmonary wards and labs (contact with contagious TB-material or TB cases) are screened every year. All other HCWs are routinely screened every third year. After exposure to a TB index case all close contacts were examined within the scope of contact investigation [6]. In accordance with the national guideline the Interferon-gamma release assay (IGRA) is used for the screening procedures. Early detection and treatment of LTBI are also recommended to prevent progression from latent TB infection to active TB [7].

So far, surveillance for LTBI in high-risk groups such as healthcare workers has been hampered by the non-specificity of the tuberculin skin test (TST) in Bacillus Calmette-Guérin (BCG) -vaccinated individuals. However, there is no gold standard test for LTBI. For about a century the tuberculin skin test (TST) was the only available test for the detection of LTBI. But the TST has its limitations, including cross-reactivity to PPD (BCG-vaccination strain) and several non-tubercular mycobacteria (NTM) infections and the need for a second visit, which often results in missed follow-ups [8].

These limitations of the TST were overcome by the development of the new T-cell based *in-vitro *Interferon- γ release assays (IGRAs), which use *M. tuberculosis-*specific antigens [3,9,10]. These assays, commercially available in the form of QuantiFERON-TB and T-SPOT.TB, have been approved by the US Food and Drug Administration and endorsed in major national guidelines for use instead of or in addition to the TST for LTBI screening [4,7,11,12]. The third generation of the QuantiFERON test (QuantiFERON^®^TB Gold In-Tube, QFT-IT) measures *in-vitro *IFN-γ production by T-cells during *in-vitro *stimulation with peptides of the *M. tuberculosis-*specific antigens of the region of difference (RD-1) ESAT-6, CFP-10 and TB7.7. These antigens are not shared by any of the BCG vaccine strains nor by the more common species of NTM (e.g. *M. avium*) [13,14]. Available evidence reviewed elsewhere [3,13,15] suggests that these IGRAs have higher specificity and at least an equal level of sensitivity as the TST and are unaffected by previous BCG vaccination and most NTM. Therefore this test reduce the risk of these tests overestimation due to cross-reactions with BCG vaccination or exposure to environmental mycobacteria [15].

So far, several systematic investigations of LTBI in HCWs using TST and IGRA have been published [16-18], showing a high proportion of TST-positive/IGRA-negative results, which is most likely explained by BCG vaccination. A couple of studies had estimated the prevalence of LTBI in HCWs in low-incidence countries with the new *in-vitro *tests [15,18-26]. These prevalence rates are much lower than those assumed for German HCWs so far. The observed previous prevalence rate in German HCWs with the non-specific TST was 24% [27].

It was the aim of our study to describe the prevalence in employees in the healthcare sector and the putative risk factors for LTBI.

## Methods

### Study design and setting

A cross-sectional study was conducted in 14 different kinds of hospitals in Germany. One of the clinics is a specialist lung disease clinic and the other clinics are general hospitals. Some of them also have special infection wards. The study population consisted of people employed in the healthcare sector tested between December 2005 and May 2009, either in the course of a contact tracing or in serial testing of TB high-risk groups in accordance with German occupational safety and health legislation (OSH) by occupational physicians. A total of 2028 employees in the healthcare sector were enrolled in the study. Of these, two smaller subgroups were already described in two studies by Nienhaus et al. (n = 261) [26] and Schablon et al. (n = 270) [24]. There were no exclusion criteria for study participants. The participants who had previous TB history were not excluded. The small sample size of 1.2% of participants with previous TB history did not affect the prevalence of LBTI in this study population:

The study protocol was approved by the ethics committee of the Hamburg Medical Council. All individuals gave their written informed consent prior to their inclusion in the study.

### Diagnostic methods

For the IGRA, the QuantiFERON-TB Gold In-Tube test was used (Cellestis Limited, Carnegie, Australia). This whole-blood assay uses overlapping peptides corresponding to ESAT-6, CFP-10 and a portion of tuberculosis antigen TB7.7 (Rv2654). Stimulation of the antigenic mixture occurs within the tube used to collect the blood. Tubes were incubated at 37°C overnight before centrifugation, and INF-γ release was measured by ELISA in accordance with the manufacturer's protocol. The QFT-IT was performed according to the manufacturer's instructions which consider a result to be positive if the IFN-γ response of TB antigen minus Nil was ≥ 0.35 UI/ml. The BCG vaccination status was confirmed by medical records or the presence of vaccination scars.

For the TST the intradermal Mendel-Mantoux test (two tuberculin units, 0.1 ml purified derivate (PPD) RT 23, Staten Serum Institute, Copenhagen, Denmark) was used. Older TST results were conducted with the multipuncture test (GT 10, Behring Tuberculin). A TST with indurations >5 mm was considered positive according to the German national guidelines [28].

### Questionnaire items

Information on the following variables was collected using a standardised questionnaire: age, gender, reason for testing, occupational exposure to TB, time of occupation in the healthcare sector, family history of TB, place of birth, prior TST, job title, workplace, chest radiographic findings and BCG vaccination. BCG vaccination was assessed through the individual vaccination register or based on scars.

All participants with a positive QFT-IT were offered a clinical and radiological examination to rule out active TB. Treatment of LTBI with isoniazid for 9 months was proposed in accordance with the German recommendations.

### Statistical analysis

Data analysis was performed using SPSS, Version 14 (SPSS Inc., Chicago, Illinois). Chi-square tests were used for categorical data. Adjusted odds ratios (OR) and 95% confidence intervals (CI) for QFT-IT, depending on different putative predictive variables, were calculated using conditional logistic regression. Model-building was performed backwards, using the chance criteria for variable selection [29]. There was a strong correlation between age and duration of employment. Therefore, we refrained from analysing both variables simultaneously.

## Results

### Study population

The study population comprises 2028 HCWs. 24 HCWs (1.2%) were excluded from the analysis due to indeterminate QFT-IT results. The majority of the participating HCWs (77.2%) were female and the mean age was 38.6 years (SD ± 11.5). A history of BCG vaccination was recorded for 45.2% of the participants. 16.8% of the study population were born outside Germany or had a history of migration (Table 1). Most of the foreign-born participants came from former Soviet Union (NIS) states and Eastern Europe (12.5%), 1.5% came from Western European countries like Spain, France, Sweden, Portugal or Switzerland. 1.3% were born in Asia, e.g. the Philippines, Thailand or India, and 1.0% of the participants immigrated from Africa or South America (no table). In the subgroup of participants from the former Soviet Union/Eastern Europe, a positive IGRA was observed in 15.9% (40/251), in the subgroup born in Western Europe the prevalence of a positive IGRA was 26.7% (8/30) and the highest prevalence of 37% was found in the subgroup born in Asia (10/27) (no Table). However, the differences were not statistically significant.

**Table 1:** Description of study population

Variable	N = 2004	%
**Age (years)**		

> 25	**253**	**12.6**

25-35 years	**481**	**24.0**

35-45 years	**571**	**28.5**

45-55 years	**507**	**25.3**

> 55 years	**192**	**9.6**

**Gender**		

Female	**1548**	**77.2**

Male	**456**	**22.8**

**Country of birth**		

Germany	**1667**	**83.2**

Foreign-born	**337**	**16.8**

**TB in own history**		

Yes	**24**	**1.2**

No	**1980**	**98.8**

**BCG vaccination**		

Yes	**905**	**45.2**

No	**1099**	**54.8**

**Profession**		

Nurse	**1087**	**54.2**

Physician	**281**	**14.0**

Trainee	**110**	**5.5**

Therapist	**66**	**3.3**

Administration	**92**	**4.6**

Ancillary nursing staff	**30**	**1.5**

Cleaning staff	**79**	**3.9**

Social workers/auxiliary	**102**	**5.1**

Radiology staff	**28**	**1.4**

Laboratory staff	**70**	**3.5**

Other	**59**	**2.9**

**Workplace**		

Admission ward	**138**	**6.9**

Infection ward	**303**	**15.1**

Geriatric care	**309**	**15.4**

Radiology/Laboratory/Pathology	**176**	**8.8**

Administration	**75**	**3.7**

Intensive care	**213**	**10.6**

Clinic ward	**721**	**36.0**

Other	**69**	**3.4**

**History of TST**		

Negative TST in history	**1017**	**50.7**

Positive TST in history	**480**	**24.0**

No TST	**507**	**25.3**

54.2% of the total study population were nurses, 14.0% were physicians, and 5.5% were trainees or young professionals (physicians, medical staff, nurses, therapists). 3.5% were laboratory staff, a total of 4.6% were administration staff (including kitchen staff, secretaries, building services), 3.9% were cleaning staff and 5.1% of the participants worked as social workers and auxiliaries (including medical secretaries, volunteers) (Table 1).

Within the scope of serial examinations of high-risk groups 68.7% (n = 1376) of HCWs were screened and 137 (10.0%) of these participants were positive in the QFT-IT (Table 1). 628 employees (31.3%) were screened in the course of contact tracing after exposure to a TB index case; in this group 9.7% were QFT-IT positive (no table). We did not find any active TB cases.

**Figure 1:**
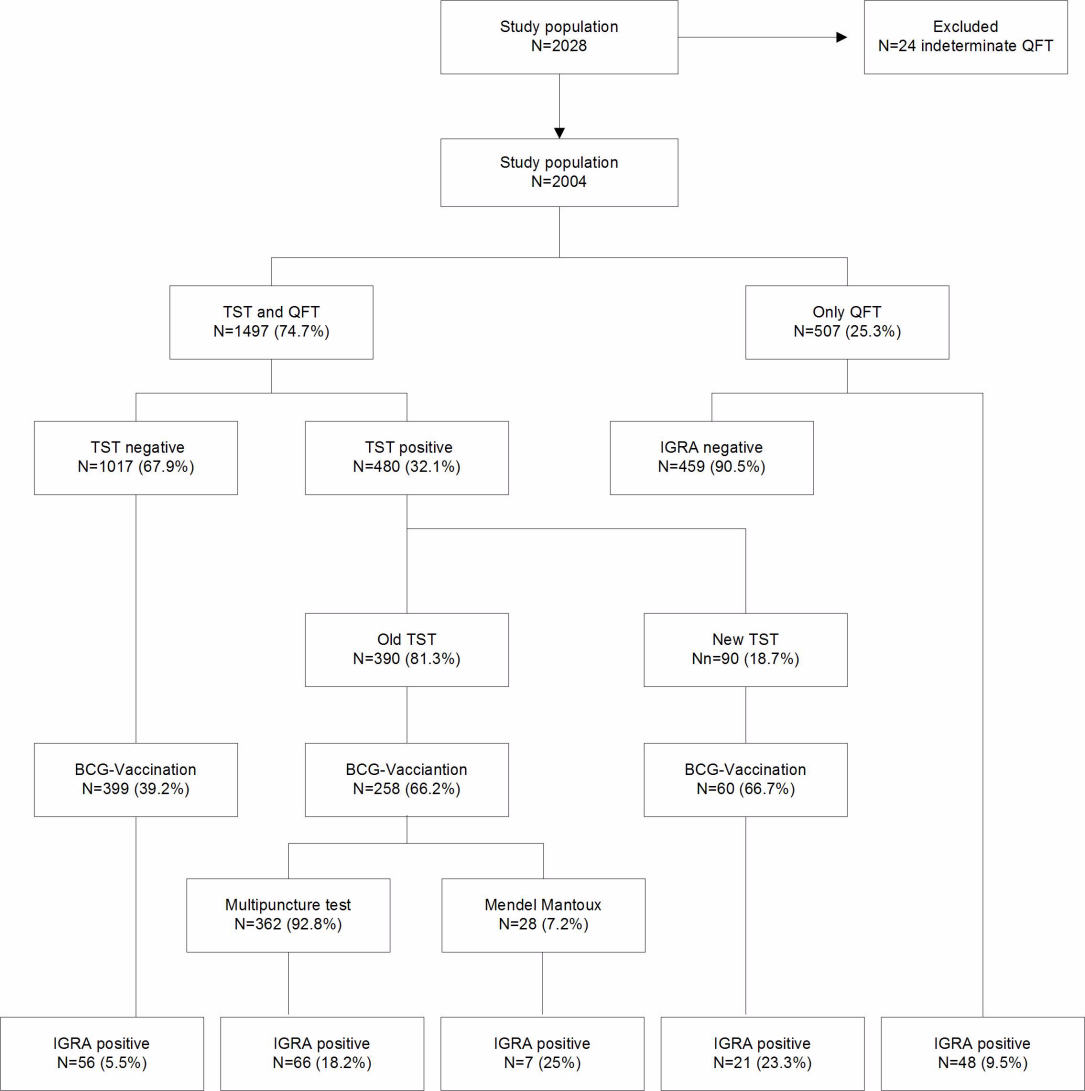
**Description of the study population depending on TST and IGRA**.

A positive QFT-IT result was observed in 198 (9.9%) participants and 480 (24.0%) participants reported a positive TST (Table 1). Of the 480 participants with a positive previous TST, 94 (19.6%) were confirmed by the IGRA Previous TST results were mostly estimated with the old multipuncture test (n = 352). Out of 507 participants with no TST in their medical history, 48 persons (9.5%) were positive by QFT-IT (Figure 1).

The prevalence of LTBI correlated with age. In the subgroup with participants under 25 years, LTBI prevalence was 1.6% and increased to 25.0% in the subgroup with participants over 55 years.

### Profession

Regarding the prevalence of LTBI in different professions we found rates of 9.7% and 9.6% in nurses and physicians. None of the individuals in the trainee group showed a positive IGRA result. The highest prevalence was observed in the group of administration staff and ancillary nursing staff (including endoscopy and bronchoscopy staff and surgery staff) (17.4% to 16.7%). In the known risk groups of technicians including radiology and laboratory staff, the observed prevalence varied from 3.6% to 11.4%. The observed prevalence in the group of cleaning staff was 11.4%. All IGRA-positive participants in this group have had a history of migration (no table).

### Workplace

The observed prevalence in the admission ward was 6.5%, compared to 8.3% in pulmonary/infectious disease wards. The highest prevalence was observed in administration staff (14.7%), geriatric care staff (12.0%) and technicians including radiology, laboratory and pathology staff (14.2%). However, the differences were not statistically significant.

### Duration of employment in healthcare sector

The prevalence of LTBI increased with the duration of employment, from 5.4% in the group with less than 5 years of employment up to 12.7% in the subgroup with more than 20 years in the healthcare sector. However, the correlation between duration of employment and age was high (r = 0.665**).

### Putative risk factors

Putative risk factors for a positive IGRA result were age (>55 years: OR14.7, 95% CI 5.1-42.1), being foreign-born (OR 1.99, 95% CI 1.4-2.8) and TB in the individual's own history (OR 4.96, 95% CI 1.99-12.3). Using the subgroup with a negative TST in their history as comparison group, the OR for those with no previous TST in their medical history was elevated (1.7, 95% CI 1.1-2.5). For those with previous positive TST results the OR was 3.5 (95% CI 2.4-4.98). No statistically significant association was observed for gender, BCG vaccination, workplace and profession (Table 2).

**Table 2:** Frequency and adjusted odds ratios (OR) and 95% confidence intervals (95% CI) for covariates associated with QFT-IT results

Covariates	QFT-IT Negative n/%	QFT-IT Positive n/%	Odds Ratio	95% CI
Age **				

> 25 years	249 (98.4)	4 (1.6)	1	

25-35 years	449 (93.3)	32 (6.7)	**3.8**	**1.3-11.2**

35-45 years	524 (91.8)	47 (8.2)	**4.4**	**1.6-12.5**

45-55 years	440 (86.8)	67 (13.2)	**7.6**	**2.7-21.4**

> 55 years	144 (75.0)	48 (25.0)	**15.1**	**5.3-43.4**

Gender**				

Female	1392 (89.9)	156 (10.1)	1	

Male	414 (90.8)	42 (9.2)	1.2	0.8-1.7

Country of birth**				

Germany	1529 (91.7)	138 (8.3)	1	

Foreign-born	277 (82.2)	60 (17.8)	**2.0**	**1.4-2.8**

TB in own history**				

No	1792 (90.5)	188 (9.5)	1	

Yes	14 (58.3)	10 (41.7)	**4.9**	**1.97-12.2**

**BCG vaccination**				

No	995 (90.5)	104 (9.5)	1	

Yes	811 (89.6)	94 (10.4)	0.9	0.7-1.3

**Profession**				

Physicians	254 (90.4)	27 (9.6)	1	

Nurses	982 (90.3)	105 (9.7)	0.94	0.6-1.6

Administration staff	146 (85.4)	25 (14.6)	1.1.	0.5-2.2

Technicians and special ward staff	175 (90.2)	19 (9.8)	0.7	0.3-1.5-

Unknown	51 (86.4)	8 (9.9)	1.3	0.5-3.3

Social workers/auxiliaries	198 (93.4)	14 (6.6)	0.8	0.4-1.8

**Workplace**				

Other clinic ward	653 (90.6)	68 (9.4)	1	

Unknown	64 (92.8)	5 (7.2)	0.9	0.3-2.4

Admission ward	129 (93.5)	9 (6.5)	0.7	0.2-2.3

Infection ward	278 (91.7)	25 (8.3)	1.3	0.4-3.8

Geriatric care	272 (88.0)	37 (12.0)	1.7	0.6-4.9

Technicians	151 (85.8)	25 (14.2)	2	0.6-6.4

Administration	64 (85.3)	11 (14.7)	1.7	0.5-5.7

Intensive care/surgery room	195 (91.5)	18 (8.5)	1.1	0.9-3.3

Previous TST in medical history**				

Negative TST	961 (94.5)	56 (5.5)	1	

Positive TST	386 (80.4)	94 (19.6)	**3.5**	**2.4-4.99**

No TST	459 (90.5)	48 (9.5)	**1.7**	**1.1-2.5**

** The final multivariate logistics model includes the variables age, gender, TST in own history, TST history and country of birth.

Thus, so far no active TB has been diagnosed in any of the healthcare employees who had a positive QFT result during the study period.

## Discussion

To the best of our knowledge, our study is the largest study to investigate the performance of the QFT-IT when screening healthcare staff in low-incidence countries like Germany. In this study we determined a low prevalence of positive QFT-IT results of 9.9%. This prevalence was substantially lower compared to the findings assumed in the past with the TST (24%)[27]. HCW with appositive TST were more often BCG vaccinated than those with a negative TST (66.2% versus 39.2%). Therefore the discrepancy is most likely due to the BCG-vaccination.

In a further analysis of smaller subgroups of our study population, Nienhaus et al. investigated 261 HCWs from different types of hospitals who are routinely screened for TB as stipulated by German OSH legislation using the QFT-IT and TST with a cut-off >5 mm. The prevalence of positive QFT-results was 9.6% [26]. Schablon et al. investigated the prevalence of LTBI in a hospital for pulmonary diseases (in the northern part of Germany) which was also included in our study population. They found a prevalence of positive QFT-IT in 7.2% of the participants[24].

In line with this, Ringshausen and colleagues reported in another study a prevalence of LTBI of 9.1% among healthcare workers during a contact investigation in a hospital after close contact with a smear-negative index case [23]. In addition, Stebler et al. [22] investigated the prevalence of LTBI among employees at the hospital of Berne using the IGRA. A positive IGRA was carried out for 59/777 HCWs (7.6%). A recent Australian study found comparable results for the frequency of positive QFT-IT (6.7%) [18]. Harada et al. also observed the performance of the QFT-G for LTBI detection by testing 332 HCWs in a Japanese general hospital and found a prevalence of positive QFT-G of 9.9% [20].

In contrast to these findings, in the study among 95 HCWs working in departments of radiology, Barsegian et al. observed a prevalence of LTBI of 1% using the T-SPOT [21]. Also, Soborg and colleagues [19] investigated the prevalence at two departments of infectious diseases in Copenhagen using QFT- TB in 139 HCWs. 34 HCWs were office staff without routine patient contact and 105 HCWs had direct patient exposure. The observed prevalence in this study population was also 1% compared to 34% with TST with a cut-off >12 mm. The positive QFT-TB rate was much lower than the estimated prevalence in our study.

In a French study Tripodi and colleagues [30] compared the performance of the TST and IGRA in French HCWs when using a high cut-off for the TST (>10 mm). 18.9% of the HCWs with recent contact to an acid-fast bacillus (AFB) -positive TB case were positive in the QFT. The rate of positive QFT is lower than the one observed in Portuguese HCWs (32.6%) [31] but higher than the one (10%) observed in German HCWs [23,24,26], which corresponded with a higher TB incidence in the Portuguese general population.

In our study none of the individuals in the trainee group showed a positive IGRA result. This finding suggests that LTBI risk is very low at the beginning of the training period in the various occupations (nurses, medical students, physical therapists). Similar to our findings, Chee et al. examined 270 medical students in Singapore in their final year of study. A positive T-Spot was found in 4.3% of the students. But this study was carried out in a country with intermediate tuberculosis incidence and it determined the prevalence at the end of training [32]. Therefore, the prevalence is somewhat higher than in Germany.

Another aim of our study was to determine putative risk factors for LTBI. We found that LTBI was associated with age (<25 years 1.6%, >55 years 25%), foreign birth and previous positive TST or no previous TST. The higher prevalence in older HCWs was also observed in other studies [20,26,33,34] and might be due to a cohort effect or the longer time at risk. These findings suggest that the IGRA describes the age dependency of LTBI prevalence in the German population very well. The association between foreign birth and LTBI was also observed on earlier analysis of a smaller subgroup of our study population published by Nienhaus et al. [26], but due to the smaller sample size the risk was not statistically significant.

We found no association between a positive QFT-IT and different kinds of occupations and workplaces. Rotation of the staff is an explanation for the lack of any association. We do not know how long the individuals were employed in a specific work area. In the course of their career, physicians and nursing staff work in various wards and hospitals; this makes it hard to attribute a positive test result to a specific work area. Furthermore, our cross-sectional design might dilute the expected association. We found no explanation for the high rate of positive QFT-IT results in the subgroup of the administration staff. We examined potential risk factors, e.g. age, migration, reason for testing (contact investigation), but no association for these putative risk factors was found. It should also be born in mind that our data are derived from routine screening of exposed HCWs. Therefore, even the seemingly unexposed group "administration" is not really unexposed.

Torres et al. investigated Portuguese HCWs with the TST and QFT-IT, and obtained findings similar to ours. Surprisingly, neither risk assessment (workplace) nor profession was associated with a positive TST or IGRA [31].

In contrast to our results, three biological studies showed a job-related exposure to TB for employees in the healthcare sector. Firstly, a molecular biological fingerprint study in Germany observed that the risk of active TB for HCWs is not higher compared to the general population. However, when the disease occurred, TB infection is most probably due to occupational exposure. In 8/10 HCWs who developed TB the infection was a consequence of occupational exposure. Secondly, Ong et al. found genotyping or epidemiological evidence of job-related transmission in at least 32% of HCWs [35]. And thirdly, de Vries and colleagues determined which TB cases among HCWs were caused by infections acquired at the workplace. Of a total of 101 TB cases, the infection pathways of 67 cases could be established; 42% were due to infection at work [36]. The majority of work-related active TB cases occurred when the infection risk was not suspected and preventive measures were not taken.

In a recent review occupational risk factors were linked to working in internal or respiratory medicine wards, duration of employment in the healthcare sector and TB exposure [3]. So far, the occupational risk of LTBI of different occupations has been investigated in several conventional studies. Most of these studies showed a significant increase of >2 in the relative risk for nurses. Several studies of varying quality examined the infection risk for physicians. Because of the inadequate data it is difficult to evaluate the LTBI risk in different medical specialties; the results do not indicate an increased infection risk for physicians. Only pathologists bear an increased LTBI risk [1].

In addition, two recently published incidence studies of Italian and Thai HCWs detected a higher risk of LTBI for specific occupational groups and units. One was a study with a homogeneous population of healthcare workers in a low-incidence area in Italy. Franchi and colleagues [37] found a low prevalence rate (6%). Working in microbiology (OR 4.16, 95% CI 1.27-13.6), dialysis/nephrology (OR 2.52, 95% CI 1.36-4.65), gynaecology/obstetrics (OR 2.46, 95% CI 1.24-4.86) and age >47 years (OR 1.98, 95% CI 1.14-3.46) were significant predictors for TB infection [30]. The second study investigated the association of workplace and the recent onset of LTBI in HCWs in a university hospital in Thailand. Working at in- and outpatient units and a history of tuberculosis exposure in the past year were significant predictors for tuberculin conversion [38]. However, these two studies were hampered by using the TST for LTBI diagnosis.

Another Italian study observed the prevalence among 115 HCWs tested simultaneously with both the TST and the IGRA. Overall, 53% of the individuals were TST-positive, 36.5% according to T-SPOT.TB and 25.2% according to QFT. An association between positives test results and an increased occupational risk of exposure to Mycobacterium tuberculosis was observed. Nurses and nurse assistants were more likely to be QFT-positive than physicians [39]. Demkow and colleagues found a higher risk of acquiring LTBI for TB lab workers (50%) and TB ward staff (34%) [40]. In our study the prevalence of LTBI in lab workers was lower (11.4%), but we could not distinguish between TB lab and non-TB lab.

In Germany all HCWs with a positive TST obtained chest x-rays after contact with a TB-index case or otherwise every third year. After the introduction of the IGRA only HCWs with a positive IGRA obtain chest x-rays. Therefore, introducing IGRA for TB screening in Germany reduces the number of X-rays and the amount of preventive chemotherapy needed by a high percentage (from 24% to 9.9%) without missing an active case of TB.

Despite our increasing knowledge, several key questions about latent TB infection remain unanswered. Particularly, it should be noted that TST and IGRA identify an adaptive immune response against M. tuberculosis, but not necessarily a latent infection. A positive result of the diagnostic tests is primarily a measure of an immunological response to stimulation by mycobacterial antigens that should not therefore be equated with the presence of live *M. tuberculosis *in the human host. It is also uncertain how long adaptive immune response towards mycobacterial antigens persist in the absence of live mycobacteria. For these reasons, according to the recently published TBNET consensus statement regarding latent TB the term "latent infection" would at best implicate "lasting tuberculosis immune response" [41].

## Limitation

The main limitation of our study is the cross-sectional study design, as mentioned before. Because of this, changes over time cannot be taken into consideration. We could not discriminate between recent and old infections. Therefore, in spite of the big sample size of more than 2000 participants, no statement can be made regarding the risks at different kinds of workplaces and occupations in the healthcare sector.

## Conclusion

In summary, our data corroborates the results of previous studies that the prevalence of LTBI in low-incidence countries depends on age. One important result of our study is that we found no positive IGRA results among trainees in the healthcare sector. Introducing IGRA for TB screening in Germany reduced the number of X-rays and the preventive chemotherapy by a high percentage (from 24% to 9.9%) while no active TB case was missed. Serial examinations are required in order to identify different kinds of occupations or workplaces especially at risk of LTBI.

## Abbreviations

ABF: acid-fast bacillus; BCG: Bacillus Calmette-Guérin; CI: confidence interval; HCW: healthcare worker; IFN: interferon; IGRA: interferon-gamma release assay; LTBI: latent tuberculosis infection; MTB: Mycobacterium tuberculosis; NTM: non-tubercular mycobacteria; OR: odds ratio; OSH: occupational safety and health; PPD: purified protein derivate; QFT-IT: QuantiFERON^®^-TB Gold In-Tube; TB: tuberculosis; TST: tuberculin skin test; TU: tuberculin unit.

## Competing interests

The authors declare that they have no competing interests.

## Authors' contributions

RD and MH have made substantial contributions to the conception and design of the study and have been involved in revising the manuscript critically for important intellectual content. They have given final approval of the version to be published. AN has made substantial contributions to the conception and design, as well as to the analysis and interpretation of data. He has been involved in drafting the manuscript. He has given final approval of the version to be published. AS has made substantial contributions to the conception and design, the acquisition of data, as well as to the analysis and interpretation of data. She has been involved in drafting the manuscript. She has given final approval of the version to be published.

## Pre-publication history

The pre-publication history for this paper can be accessed here:

http://www.biomedcentral.com/1471-2334/10/107/prepub
